# How external and agency characteristics are related to coordination in homecare – findings of the national multicenter, cross-sectional SPOT^nat^ study

**DOI:** 10.1186/s12913-024-10751-4

**Published:** 2024-03-22

**Authors:** Nathalie Möckli, Michael Simon, Kris Denhaerynck, Diana Trutschel, Tania Martins, Carla Meyer-Massetti, Franziska Zúñiga

**Affiliations:** 1 Department of Public Health, Institute of Nursing Science, Bernoullistrasse 28, CH-4056 Basel, Switzerland; 2 Clinical Pharmacology & Toxicology, Department of General Internal Medicine, Inselspital – University Hospital Bern, CH-3010 Freiburgstrasse, Bern, Switzerland; 3 Institute for Primary Health Care BIHAM, University of Bern, Mittelstrasse 30, CH-3012 Bern, Switzerland

**Keywords:** “Delivery of health care”[Mesh], “Government regulation”[Mesh], “Health services Research”[Mesh], “Healthcare financing”[Mesh], “Home care services”[Mesh], “Nursing administration research”[Mesh], “Quality of health care”[Mesh], Coordination, “Communication”[Mesh]

## Abstract

**Background:**

Homecare client services are often distributed across several interdependent healthcare providers, making proper care coordination essential. However, as studies exploring care coordination in the homecare setting are scarce, serious knowledge gaps exist regarding how various factors influence coordination in this care sector. To fill such gaps, this study’s central aim was to explore how external factors (i.e., financial and regulatory mechanisms) and homecare agency characteristics (i.e., work environment, workforce, and client characteristics) are related to care coordination in homecare.

**Methods:**

This analysis was part of a national multicentre, cross-sectional study in the Swiss homecare setting that included a stratified random sample of 88 Swiss homecare agencies. Data were collected between January and September 2021 through agency and employee questionnaires. Using our newly developed care coordination framework, COORA, we modelled our variables to assess the relevant components of care coordination on the structural, process, and outcome levels. We conducted both descriptive and multilevel regression analyses—with the latter adjusting for dependencies within agencies—to explore which key factors are associated with coordination.

**Results:**

The final sample size consisted of 1450 employees of 71 homecare agencies. We found that one explicit coordination mechanism (“communication and information exchange” (beta = 0.10, *p* <.001)) and four implicit coordination mechanisms—“knowledge of the health system” (beta = -0.07, *p* <.01), “role clarity” (beta = 0.07, *p* <.001), “mutual respect and trust” (beta = 0.07, *p* <.001), and “accountability, predictability, common perspective” (beta = 0.19, *p* <.001)—were significantly positively associated with employee-perceived coordination. We also found that the effects of agency characteristics and external factors were mediated through coordination processes.

**Conclusion:**

Implicit coordination mechanisms, which enable and enhance team communication, require closer examination. While developing strategies to strengthen implicit mechanisms, the involvement of the entire care team is vital to create structures (i.e., explicit mechanisms) that enable communication and information exchange. Appropriate coordination processes seem to mitigate the association between staffing and coordination. This suggests that they support coordination even when workload and overtime are higher.

**Supplementary Information:**

The online version contains supplementary material available at 10.1186/s12913-024-10751-4.

## Background

Care coordination improves the quality of care and reduces repeated, unnecessary, or omitted treatments. Reducing such errors benefits not only the clients, e.g., by preventing unnecessary hospitalizations, but also the health system by reducing unnecessary costs [1–3]. Therefore, given that care is increasingly shifting from inpatient to ambulatory care [4], effective care coordination is essential. At the same time, rather than moving into residential long-term care facilities, many care-dependent older patients now opt to use homecare services [5]. For the purposes of this article, homecare is understood as professional care in the client’s own home. It commonly includes combinations of personal, medical and domestic care.

Homecare differs from institutional care not only in that the care is delivered in the client’s home. Compared to residential long-term care or hospital staff, homecare workers are quite isolated, with no “backup” team available for urgent situations. Also, client services are often distributed across several health care providers [4]. To avoid schedule conflicts, and maximize efficiency, the client, their relatives, their physicians and any other professionals and institutions involved must coordinate their work. A single client’s care network might include homecare staff, a general practitioner, various specialists, social workers, pharmacists, hospitals, and residential long-term care facilities [6].

No standard definition currently applies to care coordination. However, in a review Schultz and McDonald identified several core elements of coordination, such as several participants being involved, that participants and activities are interdependent, that participants need knowledge about each other’s roles and resources, that information exchange is needed, and that the aim is to deliver proper health care [7]. To date, studies exploring care coordination in the homecare setting have focused on general practitioners or clinics [1].

Even with a very limited number of settings, international studies show that clients commonly experience care coordination gaps. These include test results not being available (often requiring repetition of those tests), various professionals giving conflicting information, unnecessary testing, specialists not receiving vital information from general practitioners [8], different physicians prescribing interacting medications, or medical records being unavailable for scheduled appointments [9, 10]. While such problems are well-documented, little is known about how external factors such as regulations or homecare agency characteristics are related to coordination in homecare.

### The care coordination framework (COORA)

Impediments to care coordination can originate at every healthcare system level. To capture these problems systematically, we developed and used the care coordination framework (COORA). Focusing on the structural and process levels relevant to coordination in homecare settings [11], the COORA framework approaches coordination as “the extent to which work dependencies are effectively managed towards a specific goal” [12, 13]. Its operative elements include external factors, agency characteristics and coordination processes.

**Fig. 1:**
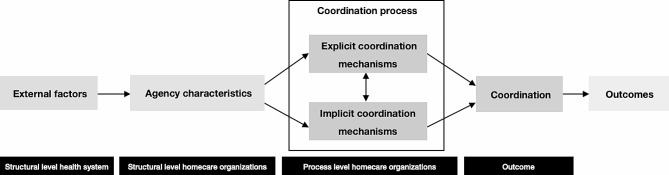
Simplified COORA framework used for this study

Managing the necessary work dependencies requires the use of *coordination mechanisms* [13, 14], i.e., approaches, methods, or tools that are available to align and synchronize work [15]. These mechanisms can be either implicit or explicit [13]. *Explicit coordination mechanisms* are behavioural. Examples include predefined plans, schedules, letters, e-mails, telephone calls, group meetings, and even defined roles. Meanwhile, *implicit coordination mechanisms* are cognitive, including shared mental models or common goals. In the literature, uses of coordination mechanisms (i.e., coordination processes) are sometimes referred to as “coordinating or coordination activities” [11]. Figure 1 schematizes the COORA framework and its connections with the various coordination elements, the examination of which provided much of the basis for this study.

### Factors that impede or enhance care coordination

Responding to a survey on how structural factors affect care coordination, home-based primary care workers in the U.S. reported that, when coordinating care, their most common barriers were financial, including patient out-of-pocket expenses, eligibility requirements, and insurance coverage [16]. Primary care providers, administrators, and insurers agreed that current financial models in the U.S. were inadequate regarding direct revenue. The shortfall impedes care coordination sustainability [17].

To search for ways to overcome such barriers Simpson et al. [18] examined institutional-level facilitators of patient-centered care and care coordination across multiple high-performing U.S. organizations. They found that these organizations shared four key foci: (i) the institutional promotion of patient-centered care and care coordination; (ii) the optimization of IT infrastructure to enhance performance and communication; (iii) the development of accountable reimbursement and incentivization structures; and (iv) the formation of organizing bodies dedicated to implementation support.

Barriers and facilitators of care coordination occur not only on the structural level, but also on that of processes. Williams, Asiedu [17] found that fragmentation of systems and communication among health care providers severely hampered care coordination. In an international review seeking care coordination enhancing factors, Morgan, Pullon [19] identified frequent opportunities for effective, informal joint communication as a key factor for successful interprofessional collaborative practice in primary care teams. They also found that multiple channels of repeated (often brief) informal joint communication were necessary to create shared knowledge, develop common goals, and facilitate shared clinical decisions. They further noted that convenient physical space and “frequent short periods [working] together” were important factors. In another review, Albertson, Chuang [1] found that three coordination facilitators were present in the majority of the reviewed coordination programs: “in-person communication with patients to coordinate care; systematic assessment of patient needs to inform subsequent care plans and interventions; and the creation of standard protocols to guide care coordination processes” (p. 80).

However, despite evidence that various structural- and process-level factors improve or hinder care coordination, we do not know how these factors are related, or which produce the most pronounced effects on coordination. In addition, information on homecare coordination processes and structures is totally absent. As effective care coordination can improve the quality of care as well as other outcomes, it is important to explore which factors influence it.

Recognizing these gaps, this study aimed to explore how, in addition to homecare agency characteristics, external factors (i.e., financial and regulatory mechanisms) are related to coordination in homecare. In accordance with this aim, guided by the COORA (care coordination) framework [11] and the literature, we formulated the following hypotheses:

1. that external factors (financial and regulatory mechanisms) are related to coordination; and specifically that, even in models where clients have to make co-payments, lower client co-payments correlate with improved coordination, as do models where agencies are reimbursed effective full costs vs. other models, as models that demand lower client outlays also tend to cover more of the cost of coordination activities;
2. that homecare agency characteristics are related to coordination—specifically, that, alongside an agency’s obligation to serve all clients and its provision of services such as oncological and palliative care, a higher percentage of RNs in the workforce, higher mean employment percentages, more years of experience per employee, higher perceived staffing, lower perceived workload, and less overtime at the employee level are all positively related to coordination, as they increase the knowledge, experience, time and other resources for coordination; and.
3. that coordination processes (including both explicit and implicit coordination mechanisms) are related to coordination. Regarding explicit coordination mechanisms, we hypothesize that the presence of clearly defined standards, the possibility of electronic data sharing possibilities, the availability of reference nurses, and the prioritization not only of routine case discussions, but also of generally clear, timely communication and information exchange are positively related to coordination. These structural arrangements allow the team to manage task dependencies and perform in concert [11]. This includes a hypothesis that implicit coordination mechanisms, including an in-depth knowledge of the health system, the availability of continuous education, clearly-defined roles, high levels of mutual respect and trust, shared perspectives, accountability and predictability are positively related to coordination. By allowing team members to anticipate one another’s tasks and their timing, these mechanisms allow them to plan accordingly [11].

## Methods

### Study design

This analysis is part of the SPOT^nat^ (quality and coordination in homecare) study, a national multicentre, cross-sectional study in the Swiss homecare setting. Detailed information on the SPOT^nat^ study can be found in the study protocol [20].

### Setting and sample

The SPOT^nat^ study included a random sample of Swiss homecare agencies. By 2021, Switzerland had 584 non-profit and 382 for-profit homecare agencies, as well as 1’421 self-employed nurses caring for around 441’000 clients. Overall, this involved roughly 59’000 care workers, a high percentage of whom were working part-time [21]. Swiss homecare is regulated at the cantonal level, which is where operating licenses are issued and the scope of professional practice defined. Its financing is drawn from three major sources: health insurers, client co-payments and residual financing by cantons, the latter two of which differ between cantons or even municipalities. For our study, agencies were stratified according to which of the country’s seven major geographic regions they served, and by whether they were for-profit or non-profit. To be eligible for inclusion, each had to employ at least ten salaried employees. For small and mid-sized agencies (10–99 employees), full samples of homecare workers were included. To reduce the study burden regarding large agencies (> 100 employees), we used random samples of 100 employees. Further details on the setting and sample, as well as the sample size calculations, can be found in the SPOT^nat^ study protocol [20]. For this sub-analysis, we included all homecare workers who (1) reported that their scope of practice included interprofessional exchanges, (2) had worked in the participating agency for at least three months, (3) worked in direct or indirect client care, (4) were at least 18 years of age and (5) were able to read and understand German, French or Italian.

### Data sources

Data were collected between January and September 2021 through investigator-developed employee and agency questionnaires completed by homecare workers and management. Each agency had three months to complete the data collection and was free to choose when during this period to distribute the paper questionnaire to their employees. The employees were given six weeks to fill out the questionnaire, which was accompanied by a stamped return envelope addressed to the research group’s institute. Questionnaires were code-numbered to match them to the participating agencies, but not to individual participants.

### Variables and measurements

The adapted COORA framework served as a basis for the modelling of our variables and guided our assessments of the various care coordination components on the structural, process and outcome levels. An overview of the measured variables, i.e., external factors, agency characteristics, coordination processes and coordination outcome variables, can be found in Table 1.

**Table 1:** Description of the independent and dependent variables

Variable	Measurement level (source of variable)	Item(s) used	Answer options/categories	Variable type / Building of scale and interpretation
**Dependent variable**				
**Coordination**				
Employee-perceived coordination	Employee questionnaire (investigator-developed)	8 items assessing how often in general: 1) relevant information is reported in a timely manner by other professionals, 2) client care activities are well aligned with other professionals, 3) there are duplicate and overlapping activities with other professionals, 4) no or no current prescriptions/ orders/ medication lists are available, 5) not all or not the right medications are available at a client’s home, 6) no one from the homecare team was involved at the discharge from an inpatient stay, 7) homecare employees do not feel sufficiently informed about a client’s condition (e.g., information is not available, only partially documented) 8) homecare employees receive important information about the client too late.	5-point Likert scale: 0 = never/almost never, 1 = rarely, 2 = sometimes, 3 = often 4 = very often for each item	Mean score over the 8 items: Cronbach’s α: 0.81 p-value χ^2^: 0.00 SRMR: 0.05 CFI: 0.86 NFI: 0.82 Higher values mean higher or better coordination, reflecting less coordination problems
**Independent variables**				
**Explicit coordination mechanisms**				
Presence of standards / guidelines for selected procedures	Agency questionnaire (investigator-developed)	5 items asking if guidelines and/or standard operating procedures (SOPs) are available for the following topics: 1) Introduction of new employees 2) Admission of a client to homecare 3) Hospital admissions 4) Emergency situations 5) Medication management	Dichotomous answer option: Yes/No for each item	Sum score over the 5 items: ranging from 0 = no SOPs at all to 5 = to all 5 topics an SOP Range VIF: 1.0–2.2 Higher values mean more standard operating procedures (SOP) are available
Electronic data sharing possibilities	Agency questionnaire (investigator-developed)	1 item asking if electronic client documentation allows for electronic data exchange of health records with general practitioners	Dichotomous answer option: Yes/No	Used as dichotomous single item
Case responsible person/managers	Agency questionnaire (investigator-developed)	3 items asking about the allocation of specific case responsibilities and case managers: 1) If the agency works with defined reference nurses that perform all nursing activities from admission to discharge or with a defined reference nurse who is responsible for the entire nursing process for a specific client 2) If the agency works with assignment of case responsible nurses 3) If the agency has trained case managers or care managers	Dichotomous answer option: Yes/No for each item	Sum score over the 3 items: ranging from 0 = no case responsibilities at all to 3 = strong emphasis on case management/responsibilities Range VIF: 1.0–1.2 Higher values mean better established reference person system
Exchange vessels	Agency questionnaire (investigator-developed)	3 items assessing if the agency has established vessels for regular exchange: 1) If the agency conducts planned case discussions for complex clients within the homecare team 2) If the agency conducts planned interprofessional/interdisciplinary case discussions for complex clients 3) If handover reports for internal client information exchange are conducted by employees	Dichotomous answer option: Yes/No for each item	Sum score over the 3 items: ranging from 0 = no regularly exchange vessels at all to 3 = all three exchange vessels in place Range VIF: 1.0–1.2 Higher values mean higher number of regularly used exchange vessels in place
Communication	Employee questionnaire (adapted from the CPAT [31])	4 items of the CPAT scale assessing communication and information exchange: 1) Assessing if relevant information relating to changes in patient/client status or care plan is reported to the appropriate team member in a timely manner. 2) Assessing if clients’ concerns are addressed effectively through regular team meetings and discussion. 3) Assessing if the team has developed effective communication strategies to share patient/client treatment goals and outcomes of care. 4) Assessing if the patient/client health record is used effectively by all team members as a communication tool.	7-point Likert scale: 1 = disagree, 2 = mostly disagree, 3 = somewhat disagree, 4 = neither agree nor disagree, 5 = somewhat agree, 6 = mostly agree, 7 = strongly agree for each item	Mean score over the 4 items Cronbach’s α: 0.83 p-value χ^2^: 0.00 SRMR: 0.01 CFI: 0.99 NFI: 0.98 The higher the values, the higher the agreement and the better the perceived communication and information exchange
**Implicit coordination mechanisms**				
Knowledge of the health system	Employee questionnaire (investigator-developed and adapted from IBenC [32])	4 items addressing how well homecare employees know the health care services in their care region: 1) Available health or social service options 2) Requirements that clients must meet in order to take advantage of the services offered 3) The area of responsibility of the other professionals/health care providers 4) Legal requirements regarding financing of health care services	Each item 5-point Likert scale answer options: 5 = Very good, 4 = good, 3 = moderate, 2 = little, 1 = not at all	Mean score over the 4 items Cronbach’s α: 0.86 p-value χ^2^: 0.976 SRMR: 0.001 CFI: 1.00 NFI: 1.00 The higher the values, the better employees rated their own knowledge of the health system
Possibility for continuous education	Agency questionnaire (investigator-developed)	3 items asking about which topics homecare agencies offer annual training for their employees (internal/external) 1) Interprofessionality and/or coordination 2) Health networks (local partners, offerings in region) 3) Legal requirements and regulations in homecare	Each item Dichotomous answer option: Yes/No	Sum score over the 3 items: ranging from 0 = no annual training possibilities to 3 = in all three domains annual training possibilities Range VIF: 1.0–1.1 The higher score meaning a higher number of training courses offered in relation to coordination
Role clarity	Employee questionnaire(COPSOQ [33, 34])	2 items of the COPSOQ asking about role clarity 1) are there clear objectives for your work? 2) do you know exactly which tasks fall within your area of responsibility?	5-point Likert scale: 4 = to a very high degree, 3 = to a high degree, 2 = in part, 1 = to a low degree, 0 = to a very low degree for each item	Mean score over the 2 items Cronbach’s α: 0.70 The higher the values, the better role clarity is rated
Mutual respect & trust	Employee questionnaire (adapted from the CPAT [31])	2 items from the CPAT assessing mutual respect and trust 1) If employees trust the accuracy of information reported among team members. 2) If team meetings provide an open, comfortable, safe place to discuss concerns.	7-point Likert scale: 1 = disagree, 2 = mostly disagree, 3 = somewhat disagree, 4 = neither agree nor disagree, 5 = somewhat agree, 6 = mostly agree, 7 = strongly agree for each item	Mean score over the 2 items Cronbach’s α: 0.56 The higher the values, the higher the mutual respect/trust and integrating conditions are rated
Accountability, predictability, common perspective	Employee questionnaire (adapted from the three Integrating Conditions scale [35])	4 items from the integrating condition scale assessing accountability, predictability and common perspective 1) If it is clear which members in your care team are responsible for completion of specific tasks. (accountability) 2) If in general the care team knows the steps necessary to address complicated situations when they arise (predictability) 3) If each member of the care team understands the steps required to complete their tasks. (predictability) 4) If the care team has a shared perspective of how each person’s work contributes to the overall goal (common perspective)	5-point Likert scale: 1 = strongly disagree, 2 = slightly disagree, 3 = neutral, 4 = slightly agree, 5 = strongly agree for each item	Mean score over the 4 items Cronbach’s α: 0.85 p-value χ^2^: 0.00 SRMR: 0.02 CFI: 0.98 NFI: 0.94 The higher the values, the higher the accountability, predictability and common perspective rated
**Homecare agency characteristics**				
Obligation to serve all clients	Agency questionnaire (adapted from Trageser, Gschwend [36])	1 item asking if the service agreement with the canton/municipalities includes the obligation to serve all clients	Dichotomous answer option: Yes/No	Used as dichotomous single item
Client characteristics	Agency questionnaire (adapted from Trageser, Gschwend [36])	1 item assessing the average care duration per client in 2020	Numeric answer option: average minutes per client	Used as numeric single item
Range of services provided	Agency questionnaire (adapted from SHURP [37])	5 items assessing if specific service offers are provided by the homecare agencies: 1) 24-hours care service, 2) Continuous night care, 3) Oncological care, 4) Palliative care, 5) Psychiatric care	Dichotomous answer options for items 1–5: Yes/No	Used as 5 dichotomous single items
Workforce	Agency questionnaire (adapted from SHURP [37])	1 item asking about the total employment percentage of the employees in the nursing and care sector at the end of the year 2020.	Numeric answer options: Working percentages of employees according to educational background Proportion of RN (or higher educated) employment percentage compared to all employees in the nursing and care sector of the agency	Used as numeric single item. A higher value represents a higher proportion of RN employment percentage over all employees
Employee characteristics	Employee questionnaire (adapted from SHURP [37])	1 item asking homecare employees about the working percentages (percentage of full-time employment)	Numeric answer option: Employment percentage	Used as numeric single item Higher values meaning higher working percentage
	Employee questionnaire (adapted from SHURP [37])	1 item asking homecare employees about the years of experience in the homecare agency	Numeric answer option: years of experience	Used as numeric single item Higher values meaning more years of experience in the homecare agency
Perceived staffing	Employee questionnaire (PES-NWI [38, 39])	3 items of the staffing and resource adequacy subscale of PES-NWI	4-point Likert scale: 1 = strongly disagree, 2 = slightly disagree, 3 = slightly agree, 4 = strongly agree for each item	Mean score over the 3 items Cronbach’s alpha 0.65 Higher values indicating better staffing and resource adequacy
Perceived workload	Employee questionnaire (NASA task-load Index [40, 41])	6 items of the NASA task-load Index	Each item 20-point analog scale answer options: low to high	Mean score over the 6 items Cronbach’s alpha 0.61 Higher values indicating higher perceived workload
Overtime	Employee questionnaire (adapted from RN4CAST [42] and SHURP [37])	1 item asking homecare employees how often they have to work overtime more than 30 min	5-point Likert scale answer option: 4 = Almost every shift, 3 = once every 2–4 working days, 2 = once every 5–7 working days, 1 = less frequently, 0 = never	Used as ordinal single item Higher values indicating more working days with overtime
**External factors**				
Reimbursement regulations of residual financing	Agency questionnaire (investigator-developed)	1 item asking on what basis the canton or municipality determined the residual financing of care costs for their agency.	Answers grouped in 4 categories: 1) Compensation of the effective full costs 2) Compensation of agency-specific and predefined costs 3) Use of standard costs, standard deficits, or maximum limits 4) others (e.g., compensation via a global budget)	Used as categorical single item
Client co-payment	Agency questionnaire (investigator-developed)	1 item asking how the amount of the patient co-payment is regulated in their canton	Answer grouped in 4 categories: 1) No patient co-payment (no payment from the client side) 2) Patient co-payment of a maximum of CHF 7.65 a day, which means that the increase can be up to 20% of the health insurance (HI) contribution or direct payment but is limited to CHF 7.65/day 3) Up to 20% of the HI contribution and upper limit of CHF 15.35/day 4) Direct contribution up to the upper limit of a maximum of CHF 15.35/day	Used as categorical single item

Note. CFI = Bentler Comparative Fit Index, COPSOQ = Copenhagen Psychosocial Questionnaire, CPAT = Collaborative practice assessment tool, HI = health insurance, IBenC = Identifying best practices for care-dependent elderly by Benchmarking Costs and outcomes of community care, NFI = Normed Fit Index, PES-NWI = Practice Environment Scale of the Nursing Work Index, RN = Registered Nurse, RN4CAST = Nurse forecasting in Europe study, SHURP = Swiss Nursing Homes Human Resources Project, SOP = standard operating procedure, SRMR = Standardized Root Mean Square Residual, VIF = Variance Inflation Factor

#### Dependent variable (outcome)

We measured *coordination* from the homecare employee perspective using eight investigator-developed items (see Table 1 for details). In line with COORA [11], the items assess whether employees perceive that coordination tasks were managed effectively so that, e.g., sequential dependencies such as transmission of information was handled in a manner that all information was available on time, or processes were aligned so that activities were not duplicated. Of these items, two were expressed positively and six negatively. The negative items were reverse-coded. All items were rated on a fully-anchored 5-point Likert scale ranging from “never/almost never” (0) to “very often” [4], with higher values indicating better coordination, i.e., reflecting fewer coordination problems. After checking the scale’s unidimensionality with principle axis factoring [22], we calculated a total score as a mean across all items. The Cronbach’s α value for our sample was 0.81 and principal axis factoring showed a shared variance of 38% with item loadings between 0.32 and 0.83 [23]; however, the model fit was rather low (cf. Table 1).

#### Independent variables

Using two agency questionnaire items formulated to assess *external factors*, we measured two homecare financial and regulatory mechanisms: (1) reimbursement regulations relevant to residual financing (costs covered by public authorities) and (2) regulation of client co-payments.

*Homecare agency characteristics* were assessed using agency and employee questionnaires. In addition to the presence of the obligation to serve all clients (i.e., the existence of a service agreement with a municipality or a canton in which the agency is bound to accept all clients in a given area without the possibility to deny service), we assessed client characteristics, the range of services provided, the workforce structure, employee characteristics and the work environment, as well as the allocation of time and other resources.

For *homecare agency coordination processes*, we assessed both explicit and implicit coordination mechanisms in the domains of programming, communication and cognition.

##### Latent variable construction

We first created scores and indices for the latent variables to be evaluated on the framework’s process level (i.e., explicit and implicit coordination mechanisms). Based on the content and the available items, we differentiated between formative and reflective indicators from a measurement perspective: indicators are either reflective, i.e., they are caused by the latent variable or formative, i.e., they cause the latent variable [24–26].

For the six reflective latent variables, i.e., “communication and information exchange,” “knowledge of the health system,” “role clarity,” “mutual respect and trust,” “accountability, predictability and common understanding,” and “coordination,” we conducted confirmatory factor analyses using the R software “lavaan” package [27]. We evaluated the model fit with a chi-square test, standardized root mean square residual (SRMR), Bentler Comparative Fit Index (CFI) and Normed Fit Index (NFI) (Table 1). As an indication of a good model fit, we expected non-significant chi-square test results with an α- level (for significance) set at 0.05, an SRMR value below 0.05, a CFI greater than 0.95 and NFI values greater than 0.90 [28]. In addition, we calculated the Cronbach’s alpha for all scales used to check internal consistency [23]. If model fit was acceptable for the reflective latent variables, we applied a second step: we calculated the mean scores, which we then used as independent variable values for the regression analysis.

For the formative latent variables, we calculated a variable score (i.e., an index) as sums of their indicators. For the measurement model assessment of these indices, we calculated the variance inflation factor (VIF) using the R “car” package [29] to check for indicator collinearity. All yielded acceptable results, i.e., values ≤ 3 [30] (Table 1). In our model, all five formative latent variables—“presence of standards / guidelines for selected procedures,” “possibility for continuous education,” “electronic data sharing possibilities,” “homecare team leaders/ case managers,” and “exchange vessels”—were measured on the agency level.

A detailed description of all the independent variables assessed can be found in Table 1. An English version of the questionnaires with the items used in this study can be found in Appendix A.

#### Demographic characteristics

For homecare agencies, we assessed each agency’s size (number of full-time equivalent employees), number of clients and hours of care provided in 2020, ownership (non-profit, for-profit), urbanicity (rural, suburban, urban) and language region (German, French, Italian).

For the employees, we assessed age, gender (male, female, non-binary), employment percentage (i.e., the proportion of a full-time workload the employee is working), years of experience in the current homecare agency, and educational background. We divided these data into two groups: (1) registered nurses (RNs), holding a Master’s or Bachelor’s degree or at least a 3-year education with a diploma; and (2) nursing and care staff with lower levels of nursing education, i.e., licensed practical nurses, certified nurse assistants or nurse aides. This group also included administrative staff, other care professionals, staff with client contact, and students/trainees.

### Data analysis

Descriptive statistics were performed to check data distribution and ceiling or floor effects, as well as to identify outliers and missing values for all variables used within the analysis. Descriptive results include frequencies and percentages (%) for categorical variables, as well as the mean (m) and standard deviation (SD) for each continuous variable.

To explore the relationships between external factors, homecare agency characteristics (structures and processes) and the degree of coordination achieved, we conducted multilevel regression analyses with the R “lme4” package [43]. To run the models, we disaggregated the agency level data to the employee level, meaning each employee was assigned a corresponding agency value. Because the theoretical framework suggests that variables are positioned in a causal chain (along with possible mediation effects), the analysis involved multiple steps. In each step, the agencies were included as random intercepts in a multilevel model. This was necessary to adjust for covariance structures within the nested design, since the intraclass correlation (ICC [1] = 0.10; CI: 0.06; 0.16) indicated inter-agency dependencies [44].

A sequential inclusion process to test several multilevel regression models was conducted. First, we modelled coordination (dependent variable) using the coordination process variables (independent) through a multilevel regression. Second, agency characteristics were added as additional independent variables to the model. Finally, we added the external factors.

If mediation of more distal variables through proximal variables occur, this should become visible by the fact that collinearity appears in later steps, meaning that (part of) the relationship of possible significant coordination process variables with coordination were explained [45]. Therefore, we compared the models to detect agency characteristics’ and/or external factors’ mediating effects on coordination.

We then ran post-hoc regression analysis using the dependent agency characteristics and external factors as independent variables. This allowed us to explore possible collinearities and to ensure that existing relationships were not masked by variables on the same causal path as the outcome variable of interest (cf. Appendix B).

We also calculated VIFs for all models.

Furthermore, we used Nakagawa’s R^2^ with the R “performance” package to evaluate each model’s explanatory power [46]. While the *marginal* R^2^ takes the variance of only the fixed effects into account, the *conditional* R^2^ takes both fixed and random effects into account [47].

The results of the regression models are presented with the coefficient estimates ($\beta$), alongside their 95% confidence intervals (CIs). A p-value of < 0.05 was considered significant. For this analysis we only included complete cases; therefore, missing data sets were deleted listwise. We conducted a sensitivity analysis by running a regression model with a complete data set with only process variables (implicit and explicit coordination mechanisms). This showed whether missing values changed the model’s conclusion (Appendix C). Data analyses were conducted with the R 4.2.1 software [48].

## Results

A total of 3223 employees (response rate: 73.6%) of the 88 participating homecare agencies completed the questionnaire. After we applied the inclusion criteria for our analysis, only the 1784 employees who stated that exchanges with other professions fell within their scope of practice remained in the sample. A median of 13 employees per agency participated (interquartile range: 7–27 participants). After removing incomplete answer sets, a final sample size of 1450 employees of 71 homecare agencies remained for the statistical analyses. The exclusion of 17 homecare agencies was due to missing values in “agency characteristics” and “external factors” (cf. Table 2). However, the conclusion of the sensitivity analysis, which included all 88 agencies, did not change.

**Table 2:** Descriptive characteristics of the sample and the dependent and independent variables

Variables	*n* (%)	Mean (SD)	Missing *n* (%)	
**Homecare agencies**	**88**			
Ownership			0	
non-profit	62 (70.5)			
for-profit	26 (29.5)			
Urbanicity			0	
Rural	39 (44.3)			
Suburban	32 (36.4)			
Urban	17 (19.3)			
Language region			0	
German	67 (76.1)			
French	14 (15.9)			
Italian	7 (8.0)			
Size				
Number of full-time equivalents (FTE)		45.6 (57.5)	0	
Total number of clients in 2020		557.2 (734.7)	3 (3.4)	
Hours of care provided in 2020		41,404 (42582.3)	2 (2.3)	
**Independent variables measured on agency level**				
Coordination Process				
*Explicit coordination mechanism*:				
Programming				
Presence of standards / guidelines (index 0–5)		3.8 (1.2)	0	
Case responsible/managers (index 0–3)		1.9 (0.8)		
Exchange vessels (index 0–3)		2.5 ()		
Electronic data sharing possibilities with physicians (yes)	22 (25.0)		0	
*Implicit coordination mechanism*:				
Cognition				
Possibility for continuous education (index 0–3)		0.7 (0.9)		
Agency characteristics				
Obligation to serve all clients (yes)	58 (65.9)		0	
Range of service				
Palliative Care (yes)	64 (72.7)		0	
Oncology care (yes)	18 (20.5)		0	
Psychiatric care (yes)	59 (67.0)		0	
24 h care service (yes)	26 (29.5)		0	
Continuous night care (yes)	28 (31.8)		0	
Average hours of care billed per client in 2020		83.0 (62.3)	4 (4.5)	
Workforce				
Percentage of RNs or higher educational background		29.7 (13.8)	9 (10.2)	
External factors				
Financial regulatory mechanisms				
Reimbursement regulations of residual financing			7 (8.0)	
Effective full costs	22 (27.2)			
Agency-specific and predefined costs	27 (33.3)			
Standard costs, standard deficits, or maximum limits	29 (35.8)			
others (e.g., compensation via a global budget)	3 (3.4)			
Client co-payment			0	
No patient co-payment		18 (20.5)		
Co-payment of a maximum of CHF 7.65 a day		34 (38.6)		
Up to 20% of the HI contribution and upper limit of CHF 15.35/day		29 (33.0)		
Direct contribution up to the upper limit of a maximum of CHF 15.35/day		7 (8.0)		
**Employees**	**1784**			
Age		44.6 (12.1)	58 (3.3)	
Gender			16 (0.9)	
Female	1625 (91.9)			
Male	140 (7.9)			
Non-binary	3 (0.2)			
Educational background			13 (0.7)	
RNs with a Master or Bachelor degree or at least a 3-year education with diploma	1085 (61.3)			
Nursing and care staff with lower education in the nursing field	686 (38.7)			
Language region			0	
German	1148 (64.3)			
French	549 (30.8)			
Italian	87 (4.9)			
**Independent variables measured on employee level**				
Coordination Process				
*Explicit coordination mechanism*:				
Communication				
Communication and information exchange (scale 1–7)		4.8 (1.3)	6 (0.3)	
*Implicit coordination mechanism*:				
Cognition				
Knowledge of the health system (scale 1–5)		3.7 (0.8)	14 (0.8)	
Role clarity (scale 0–4)		3.0 (0.7)	8 (0.4)	
Mutual respect and trust (scale 1–7)		5.3 (1.1)	6 (0.3)	
Accountability, predictability, common perspective (scale 1–5)		3.8 (0.7)	4 (0.2)	
Agency characteristics				
Employment percentage (%)		70.0 (21.3)	31 (1.7)	
Experience in agency (in years)		6.5 (6.7)	89 (5.0)	
Perceived staffing (scale 1–4)		2.9 (0.7)	21 (1.2)	
Perceived workload (scale 1–20)		10.8 (2.7)	6 (0.3)	
Overtime (single item 0–4)		0.74 (0.44)	25 (1.4)	
At least once a week	1299 (73.8)			
**Dependent variable measured on employee level**				
Employee-perceived coordination (scale 0–4)		2.52 (0.61)	7 (< 0.01)	

Note. CHF = Swiss francs, FTE = full-time equivalent posts, HI = health insurance, RN = registered nurse, SD = standard deviation

### Descriptive sample characteristics

Participating homecare agencies were predominantly non-profit (70.5%) and based in the German-speaking part of Switzerland (76.1%). They employed a mean of 45.6 full time equivalents (FTEs) (range: 4.7–318.0 FTEs). The participating homecare workers’ mean employment percentage was 70% (range: 5–100%). The participating employees were mostly female (91.9%) and had an average age of 44.6 years old (range: 18–76 years). The majority (58.3%) were RNs with nursing diplomas or higher degrees (3.0%). Table 3 shows the sample characteristics.

**Table 3:** Results of the regression analyses with employee-perceived coordination (employees *n* = 1450; agencies *n* = 71)

		Coordination regressed only with coordination process variables		Coordination regressed with coordination process & agency characteristic variables		Coordination regressed with coordination process agency characteristic & external factors variables
		$\beta$ [95% CI]		$\beta$ [95% CI]		$\beta$ [95% CI]
**Coordination Process**						
**Explicit coordination mechanism** (Programming & Communication)						
Presence of standards / guidelines		0.00 [-0.03; 0.03]		0.01 [-0.03; 0.04]		0.01 [-0.02; 0.04]
Case responsible/managers		-0.03 [-0.09; 0.02]		-0.01 [-0.07; 0.05]		0.00 [-0.06; 0.05]
Exchange vessels		-0.03 [-0.09; 0.02]		-0.03 [-0.08; 0.02]		-0.03 [-0.09; 0.02]
Electronic data sharing with physicians: yes		0.04 [-0.04; 0.12]		0.03 [-0.06; 0.11]		0.03 [-0.06; 0.10]
Communication and information exchange		0.11* [0.07; 0.14]		0.10* [0.06; 0.13]		0.10* [0.06; 0.13]
**Implicit coordination mechanism** (Cognition)						
Knowledge of the health system		-0.09* [-0.12; -0.05]		-0.07* [-0.10; -0.03]		-0.07* [-0.10; -0.03]
Possibility for continuous education		-0.03 [-0.07; 0.01]		-0.02 [-0.05; 0.02]		-0.01 [-0.05; 0.03]
Role clarity		0.10* [0.06; 0.14]		0.08* [0.03; 0.11]		0.07* [0.03; 0.11]
Mutual respect and trust		0.08* [0.04; 0.12]		0.07* [0.03; 0.11]		0.07* [0.03; 0.11]
Accountability, predictability, common perspective		0.21* [0.17; 0.26]		0.19* [0.15; 0.24]		0.19* [0.14; 0.24]
**Agency characteristics**						
Obligation to serve all clients (yes)				-0.14* [-0.26; -0.02]		-0.14 [-0.31; -0.02]
Range of service						
Palliative Care (yes)				-0.02 [-0.15; 0.05]		-0.02 [-0.11; 0.09]
Oncological care (yes)				-0.01 [-0.09; 0.08]		0.00 [-0.07; 0.09]
Psychiatric care (yes)				0.03 [-0.10; 0.16]		0.04 [-0.09; 0.16]
24-hour care service (yes)				0.04 [-0.07; 0.15]		0.04 [-0.09; 0.13]
Continuous night care (yes)				-0.09 [-0.21; 0.03]		-0.05 [-0.16; 0.05]
Percentage of RNs				0.00 [0.01; 0.00]		0.00 [-0.01; 0.00]
Employment percentage				0.00 [0.00; 0.00]		0.00 [0.00; 0.00]
Experience in agency				0.00 [-0.01; 0.00]		0.00 [-0.00; 0.00]
Perceived staffing				0.05* [0.01; 0.10]		0.06* [0.01; 0.10]
Perceived workload				-0.01* [-0.03; 0.00]		-0.02* [-0.03; -0.01]
Overtime				-0.05* [-0.08; -0.03]		-0.05* [-0.07; -0.02]
**External factors**						
Reimbursement regulations of residual payments (reference: effective full costs)						
agency-specific and predefined costs						0.10 [-0.01; 0.18]
standard costs						-0.04 [-0.17; 0.07]
others						-0.00 [-0.22; 0.23]
Client co-payment (reference: No co-payment)						
maximum of CHF 7.65 a day						-0.06 [-0.18; 0.03]
up to 20% of HI, with max CHF 15.35/d						-0.08 [-0.20; 0.03]
direct with max. of CHF 15.35/d						-0.05 [-0.17; 0.09]
Average hours of care per client						0.00 [0.00; 0.00]
**Second level variable**						
Homecare agencies						
Agency level (Variance [SD])		0.01 [0.11]		0.01 [0.11]		0.01 [0.10]
Residuals (Variance [SD])		0.24 [0.48]		0.22 [0.47]		0.23 [0.48]
**Effect size**						
AIC		2143.98		2183.22		2222.33
Marginal *R*^*2*^		0.328		0.363		0.367
Conditional *R*^*2*^		0.359		0.395		0.395

Note. AIC = Akaike Information Criterion, CHF = Swiss Francs, CI = Confidence Interval, HI = health insurance, RN = Registered nurse, SD = Standard Deviation,

α levels of significance = *p <.05, $\beta$ = coefficient estimate

### Description of the dependent and independent variables

The participating employees rated their perceived coordination with a mean value of 2.5 on a scale from 0 to 4, i.e., above average. Just over two-thirds (67.6%) indicated that they often/very often receive relevant information from other professionals at the right time. However, 14.5% reported that important information about clients was often/very often received too late. While 66.9% stated that care activities are often well-aligned between professionals and 12.3% stated that duplicate or overlapping activities almost never/never happen, 8.0% reported duplicate or overlapping activities happening often/very often. Regarding client transfers from hospital inpatient stays to home, 22.6% of respondents reported that often/very often no one from the homecare team was involved in the discharge process. Detailed results are shown in Appendix D.

For the independent variables, the participating employees allocated above-average values for their communication and information exchanges (m = 4.8, SD = 1.3; scale 1–7) and knowledge of the health system (m = 3.7, SD = 0.8; scale 1–5), the clarity of their roles (m = 3.0, SD = 0.7; scale 0–4), mutual respect and trust (m = 5.3, SD = 1.1; scale 1–7), and accountability/predictability/common perspective (m = 3.8, SD = 0.7; scale 1–5).

Of the 88 agencies represented, 79 worked with three or more SOPs and 29 had SOPs for all five assessed processes. Only six did not delegate case responsibilities, whereas fifteen allocated some case-administration responsibilities to nurses and trained other staff as case managers or care managers. Considering exchange vessels, over half of the agencies (*n* = 49) worked with handover reports, with the majority (*n* = 79) conducting case discussions for complex clients within the homecare team. On the other hand, 51 agencies did not offer annual training for their employees on the three assessed topics (interprofessional collaboration and/or coordination, health networks, legal requirements & regulations in homecare). Further descriptive characteristics of the dependent and independent variables are shown in Table 3 (below).

### External factors’, agency characteristics’, and the coordination process’s relationships with coordination

The results of the final analysis, which tested the full regression model (step 3 of the model), showed several significant positive associations. One explicit coordination mechanism, “communication and information exchange” ($\beta$ = 0.10, *p* <.001), and four implicit ones—“knowledge of the health system” ($\beta$ = -0.07, *p* <.01), “role clarity” ($\beta$ = 0.07, *p* <.001), “mutual respect and trust” ($\beta$ = 0.07, *p* <.001), and “accountability, predictability, common perspective” ($\beta$ = 0.19, *p* <.001)—correlated with employee-perceived coordination. Regarding agency characteristics, only three variables—“perceived staffing” ($\beta$ = -0.06, *p* <.05), “perceived workload” ($\beta$ = -0.02, *p* <.01) and “overtime” ($\beta$ = -0.05, *p* <.001)—reached statistical significance, while none of the external factors did. “Knowledge of the health system,” “perceived workload,” “perceived staffing” and “overtime” showed very weak associations. The R^2^ indicated that, in the final model, approximately 37% of the variance in employee-perceived coordination data was explained by the predictor variables. For details of the regression results, see Table 2.

The results provided in Appendix B suggest that both agency characteristics and external factors may be mediated to some degree through coordination processes. Staffing, workload and overtime were initially significant in both models but lost half the strength of their associations when the process variables were added to the model. Two variables lost their significance after adding the process variables, namely *service obligation* and *average hours of care per client*. The R^2^ values of both models, both with and without external factors, are nearly the same; therefore, the full model does not explain the data more clearly when it includes external factors as predictors than when it excludes them. And as noted, the sensitivity analysis did not change the model’s conclusion (Appendix C).

## Discussion

The aim of this study was to explore how, regarding homecare agencies, both external factors and internal structures and processes are related to care coordination as an outcome, i.e., to explore the extent to which work dependencies are effectively managed to reach a specific client outcome. On the process level, in line with our third hypothesis, we found that communication, role clarity, mutual respect and trust, as well as accountability, predictability, and common perspective correlate positively with employee-perceived care coordination. While the correlation was relatively weak, employee knowledge of the health system correlated negatively with employee-perceived care coordination. I.e., better healthcare system knowledge was associated with lower perceived care coordination ratings. One possible explanation is that respondents with more healthcare system knowledge recognized more coordination shortfalls, leading to more critical appraisals. Whatever the reason, the importance of knowing and working with the healthcare system to connect patients with the care they need has been reported by previous studies [49, 50]. This correlation cannot be ignored.

On the structural level, we found that overtime and higher perceived workload correlated negatively and higher perceived staffing correlated positively with employee-perceived care coordination. While these correlations supported our second hypothesis, they were marginal. We did not confirm our first hypothesis, i.e., the evidence does not indicate relationships between external factors and coordination.

However, one major finding of this study is that, while almost all implicit mechanisms were significantly associated with perceived care coordination, explicit mechanisms other than communication were not. Admittedly, this finding might be biased by the level at which the relevant assessments were made. Whereas all significant correlations were measured at the employee level, all insignificant mechanisms were measured at the agency level. Given that only 71 agencies were included in the analysis, the smaller sample’s variability may have been inadequate to detect significant differences. Another plausible interpretation of this finding is that, alongside the explicit mechanism of communication, implicit mechanisms are most influential regarding successful coordination.

As for explicit coordination mechanisms, previous studies have confirmed the importance of communication and information exchange. Qualitative research has identified it as a key factor for successful coordination [18, 51–54]. Mohr, Benzer [55] highlighted the value of inter-team communication in caring for complex clients. In our study group, considering that almost a quarter of homecare workers reported that often/very often no one from the homecare team was involved in the client transfers home from inpatient stays, while over one-tenth reported often/very often receiving important information too late, there is considerable room for improvement.

To the best of our knowledge, this is the first study to examine implicit coordination mechanisms in the homecare setting. Its results will support previous qualitative indications that implicit mechanisms, e.g., role clarity [53, 56], mutual respect and trust [17, 57] and accountability, as well as a common perspective [18, 53], contribute essentially to successful coordination. Gittell [58], who developed the concept of relational coordination, indicated that the explicit mechanism of communication (i.e., frequent, timely, accurate and problem-solving communication), and the implicit mechanisms tied to relationships (i.e., shared goals, shared knowledge, and mutual respect) are essential elements of coordination. Gittell’s concept of relational coordination does not distinguish between coordination as a process and as an outcome; nevertheless, as it focuses on interpersonal relationships, we can support the conclusion that the above-named mechanisms of communication and relationships are positively associated with improved coordination not only in hospitals [59] but also in homecare. In addition, relational coordination has been linked to improved quality outcomes regarding, e.g., nursing care goals [60], better chronic care delivery [61], better patient perception of care [62] and higher patient satisfaction [63]. In addition, Cramm, Hoeijmakers [64] reported both that comprehensive care delivery demands strong connections between all involved health and social care professionals and that homecare nurses play an important role in strengthening those connections. Here, opportunities for face-to-face discussions in homecare—whether at conferences or workshops—can foster good relationships among colleagues [65].

At the agencies’ structural level, we observed that key work environment factors— perceived staffing, workload and overtime—were related to care coordination. This adds to the literature, where such variables have largely been explored in view of their relationships with quality outcomes in homecare [66–70] but have not previously been assessed in view of care coordination. As appropriate processes appear to mitigate associations between (low) staffing and coordination problems, they likely support and maintain coordination even when workload and overtime are high.

As noted above, neither of the external factors we measured correlated with care coordination; however, our model only included financial aspects and care hours per client. Building implicit coordination mechanisms is a long-term process [13]. Prerequisites to their formation include the presence of various other external factors, e.g., a sufficiently trained workforce and the provision of adequate vessels for inter-organizational connections. As these factors are time- and resource-intensive, they may not be implemented voluntarily [6]. However, they certainly warrant further exploration.

This study also served as the first empirical test of the COORA framework. By transparently mapping coordination processes and outcomes, COORA illuminates the key mechanisms and their effects on coordination outcomes. By showing this process in action, this study substantiates the usefulness of the COORA framework, which clearly differentiates between implicit and explicit mechanisms, and, most importantly, between coordination as a set of processes and coordination as an outcome.

### Limitations

This study has several notable strengths and limitations. First, its cross-sectional design does not allow causal inferences. Second, homecare settings are very location-specific, i.e., they differ considerably between countries; therefore, our findings’ transferability and generalizability are limited to Switzerland. However, the analysis is based on the COORA framework, which is firmly rooted in international literature across diverse research areas. Therefore, the framework and methodology used here should be applicable to international health care settings. Third, the timing of our data collection—during the COVID-19 pandemic—could have influenced employees’ perceptions of their work environment, particularly regarding workload and overtime. Fourth, due to pandemic-related challenges, the targeted homecare agency sample size could not be reached, reducing the reliability of our results. However, the sample of homecare employees was sufficient for our needs.

In addition, some scales and indices were investigator-developed and had not yet been validated. These included the employee-perceived care coordination scale, which showed insufficient model fit in the CFA, and the *mutual respect and trust* variable, which showed a low Cronbach’s alpha; therefore, we cannot be certain that we adequately measured the intended construct. However, our development of the scales used to measure aspects of care coordination was theoretically grounded and built upon previously-used content. In general, as proper measurements have not yet been developed and tested to measure explicit mechanisms of coordination, the items and indices used were based on peer-reviewed results and expert opinion, but had not been validated. This is a weakness.

Biases also raise some concerns. As the study design did not allow the researchers to control the environment during data collection, social desirability bias cannot be excluded. Similarly, because of the questionnaire design, some recall and common-method bias may have crept in.

Regarding our analyses, it is difficult to judge which increases in the regression coefficients used for the coordination scale are clinically meaningful. It is also possible that unconsidered factors and confounders influenced our results.

Finally, this study’s outcome of interest was employee-rated coordination. Considering that clients are equally part of the coordination process, further research should examine their perspectives on coordination (as an outcome) to assess its relationship with coordination mechanisms. This would have the added benefit of providing insights into how COORA functions as a coordination framework.

## Conclusion

This study’s results indicate that, in addition to one explicit coordination mechanism (communication), four implicit coordination mechanisms play significant roles in the process of care coordination: *role clarity*, *mutual respect/trust, accountability*/*predictability/common perspectives*, and *knowledge of the health system*. We recommend that homecare administrators reflect on which coordination mechanisms are strongest and weakest in their contexts. However, they should also be aware that, especially regarding communication and information exchange, achieving high-quality coordination (as an outcome) may require the addition of explicit mechanisms that facilitate these processes. Developing successful strategies on how implicit mechanisms and communication could be strengthened demands the involvement of the entire care staff.

Additionally, while the COORA framework clearly provided us with very useful guidance for this study, it requires further testing. Tools to measure the various implicit and explicit mechanisms should also be developed. Finally, building a deeper understanding of the coordination process will require an examination of whether explicit mechanisms can be used to build implicit mechanisms.

### Electronic supplementary material

Below is the link to the electronic supplementary material.

Supplementary Material 1

Supplementary Material 2

Supplementary Material 3

Supplementary Material 4

## Data Availability

The datasets generated and/or analyzed during the current study are not publicly available due to the sensitivity of the data, but are available from the corresponding author on reasonable request.

## Abbreviations

AIC: Akaike Information Criterion
CFA: Confirmatory Factor Analysis
CFI: Bentler Comparative Fit Index
CHF: Swiss Francs
CI: Confidence Interval
COORA: Care Coordination framework
COPSOQ: Copenhagen Psychosocial Questionnaire
CPAT: Collaborative Practice Assessment Tool
EKNZ: Ethics Committee of Northwestern and Central Switzerland
FTE: Full-Time Equivalent posts
HI: Health Insurance
IBenC: Identifying best practices for care-dependent elderly by Benchmarking Costs and outcomes of community care
ICC: Intraclass Correlation Coefficient
NA: Not Applicable
NFI: Normed Fit Index
PES-NWI: Practice Environment Scale of the Nursing Work Index
RN: Registered Nurse
RN4CAST: Nurse forecasting in Europe study
SD: Standard Deviation
SHURP: Swiss Nursing Homes Human Resources Project
SOP: Standard Operating Procedure
SPOT^nat^: Spitex Koordination und Qualität– eine nationale Studie (homecare coordination and quality - a national study)
SRMR: Standardized Root Mean Square Residual
VIF: Variance Inflation Factor
