# Supplementary material for: How external and agency characteristics are related to coordination in homecare – findings of the national multicenter, cross-sectional SPOTnat study
Source: BMC Health Serv Res. 2024 Mar 22;24:367. doi: 10.1186/s12913-024-10751-4 (PMC10960419; doi:10.1186/s12913-024-10751-4)
Supplement: Supplementary file 3 — Supplementary Material 3 [file 12913_2024_10751_MOESM3_ESM.docx]

**Appendix C. Sensitivity Analysis**

We conducted a sensitivity analysis by running the regression model for coordination with coordination process variables on the dataset with complete cases only for these variables. For these variables, the complete data set consisted of n= 88 agencies and n= 1748 employee responses

|  |  | Coordination regressed only with coordination process variables (n=1748) |
| --- | --- | --- |
|  |  | $\beta$ [95% CI] |
| **Coordination Process** |  |  |
| **Explicit coordination mechanism (**Programming & Communication) |  |  |
| Presence of standards / guidelines |  | 0.00 [-0.03; 0.03] |
| Case responsible/managers |  | -0.04 [-0.09; 0.01] |
| Exchange vessels |  | -0.02 [-0.07; 0.04] |
| Electronic data sharing with physicians: yes |  | 0.02 [-0.07; 0.10] |
| Communication and information exchange |  | 0.12*** [0.09; 0.15] |
| **Implicit coordination mechanism** (Cognition) |  |  |
| Knowledge of the health system |  | -0.09*** [-0.12; -0.06] |
| Possibility for continuous education |  | -0.02 [-0.06; 0.01] |
| Role clarity |  | 0.10*** [0.06; 0.13] |
| Mutual respect and trust |  | 0.07*** [0.03; 0.10] |
| Accountability, predictability, common perspective |  | 0.21*** [0.17; 0.25] |
| **Second level variable** |  |  |
| Homecare agencies  Agency level (Variance [SD]) Residuals (Variance [SD]) |  | 0.02 [0.12] 0.24 [0.49] |
| **Effect size** |  |  |
| AIC |  | 2585.04 |
| Marginal *R^2^*  Conditional *R^2^* |  | 0.331 0.371 |

**Note.** AIC = Akaike Information Criterion, CI = Confidence Interval, SD= Standard Deviation, α levels of significance = *p < .05, **p < .01, ***p < .001, $\beta$ = coefficient estimate
