# Supplementary material for: How external and agency characteristics are related to coordination in homecare – findings of the national multicenter, cross-sectional SPOTnat study
Source: BMC Health Serv Res. 2024 Mar 22;24:367. doi: 10.1186/s12913-024-10751-4 (PMC10960419; doi:10.1186/s12913-024-10751-4)
Supplement: Supplementary file 4 — Supplementary Material 4 [file 12913_2024_10751_MOESM4_ESM.docx]

**Appendix D. Employee-perceived Coordination**

Table S1: answer options percentage of each single item of the employee-perceived coordination.

| **Items** | **Question formulated** | **Categories*** | | | | | **Missing**** |
| --- | --- | --- | --- | --- | --- | --- | --- |
|  | In general, how often… | never / almost never | seldom | sometimes | often | very often |  |
| P56 | is relevant information reported in a timely manner by other professionals | 0.7 | 3.3 | 28.4 | 55.9 | 11.7 | 19 (0.01) |
| P58 | are client care activities well aligned with other professionals | 0.3 | 4.0 | 28.7 | 57.1 | 9.8 | 20 (0.01) |
| P59 | are there duplicate and overlapping activities with other professionals | 12.3 | 39.4 | 40.2 | 6.9 | 1.1 | 27 (0.02) |
|  | How often does it happen that… |  |  |  |  |  |  |
| P60 | not all or not the right medications are available at a client’s home | 17.3 | 39.1 | 32.7 | 8.9 | 2.0 | 100 (0.06) |
| P61 | no or no current prescriptions/ medication/ medication lists are available | 15.1 | 33 | 34.0 | 14.5 | 3.4 | 98 (0.05) |
| P62 | no one from the homecare team was involved at the discharge from an inpatient stay | 16.2 | 31.4 | 29.8 | 14.5 | 8.1 | 270 (0.15) |
| P63 | you do not feel sufficiently informed about a client's condition (e.g., information is not available, only partially documented) | 9.5 | 31.2 | 38.5 | 15.8 | 5.1 | 50 (0.03) |
| P64 | you receive important information about the client too late | 11.8 | 38.8 | 34.9 | 11.4 | 3.1 | 41 (0.02) |

*Distribution of the item categories are given in %, **the missing in numbers n (%)

| 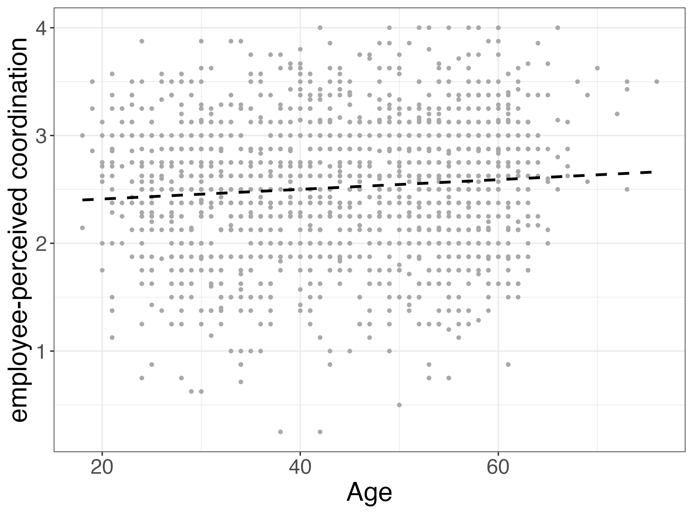 | 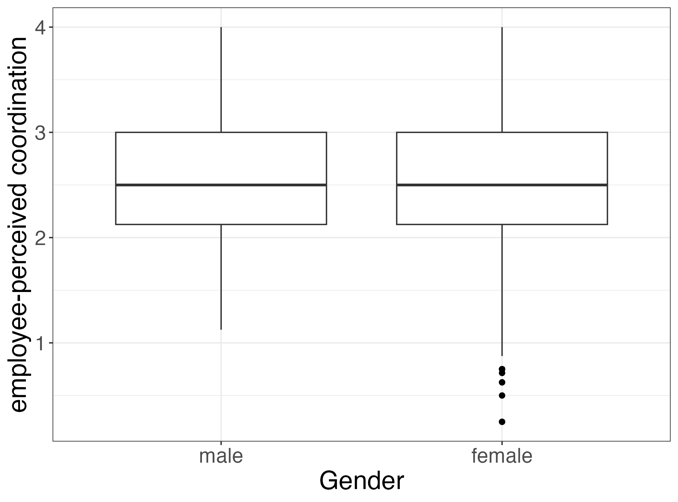 | 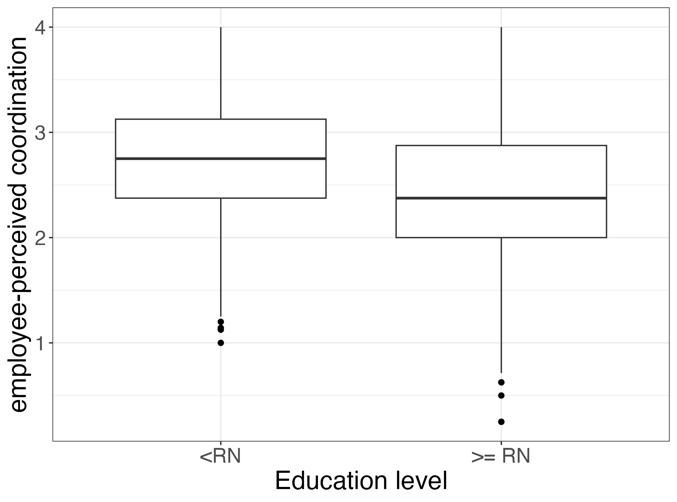 |
| --- | --- | --- |

Figure S1: Distribution of the employee-perceived coordination over age (left), age (middle) and gender (right), wherby the mean (sd) of male and female employees is 2.53 (0.59) and 2.52 (0.61), for employees with lower education in the nursing field is 2.73 (0.57) and for RNs with a Master or Bachelor degree or at least a 3-year education with diploma is 2.39 (0.6)
